# Antitumor Effects of Hesperidin and Cisplatin on Human Osteosarcoma Cells Through Inhibiting Proliferation and Inducing Mitochondrial-Mediated Apoptosis

**DOI:** 10.3390/medicina61060960

**Published:** 2025-05-23

**Authors:** Mehmet Onur Ziyadanoğulları, Mehmet Cudi Tuncer, İlhan Özdemir

**Affiliations:** 1 Department of Orthopaedics and Traumatology, Gazi Yasargil Research and Training Hospital, Health Sciences University, Diyarbakır 21070, Turkey; monurziya@gmail.com; 2 Department of Anatomy, Faculty of Medicine, Dicle University, Diyarbakır 21280, Turkey; 3 Department of Gynecology and Obstetrics, Faculty of Medicine, Atatürk University, Erzurum 25240, Turkey; ilhanozdemir25@yandex.com

**Keywords:** osteosarcoma, hesperidin, cisplatin, apoptosis, Bax

## Abstract

*Background and Objectives*: Osteosarcoma is a primary malignant bone tumor characterized by the proliferation of malignant mesenchymal cells and primarily affects children and adolescents. Hesperidin (Hes) interacts with various cellular targets and inhibits cancer cell proliferation by inducing apoptosis. However, the precise mechanisms underlying Hes-induced cell death in osteosarcoma cells remain unclear. This study aimed to investigate the effects of Hes and cisplatin (Cis) on the Bax/Bcl-2 apoptotic pathway in osteosarcoma cells. *Materials and Methods*: The human osteosarcoma cell line U2OS (Uppsala 2 Osteosarcoma) was treated with IC_50_ concentrations of Hes and Cis for 48 h. Changes in the mRNA expression levels of Bax, Bcl-2, Caspase-3, and Survivin—key regulators of apoptosis—were analyzed using quantitative real-time PCR (qPCR). The synergistic and/or antagonistic interactions of the Hes and Cis combination were evaluated using Combenefit v2.021 software (Cambridge, UK). *Results*: The dose–response curve for Hes revealed a gradual reduction in cell viability, with an IC_50_ value of 106 µM, while the IC_50_ value for Cis was 4.83 µM. The levels of the inflammatory cytokines IL-1β, TNF-α, and IFN-γ were significantly decreased in the treatment groups compared to the control (*p* = 0.01). IL-6 levels also showed a marked decrease, particularly in the Hes and Cis groups, with high statistical significance (*p* = 0.002). Treatment with Hes and Cis significantly upregulated the mRNA expression of Bax and Caspase-3, while significantly downregulating Bcl-2 and Survivin mRNA levels (*p* < 0.05). Notably, Bax expression was highest in the Hes + Cis combination group. The combination treatment exhibited enhanced cytotoxicity, especially at higher concentrations, indicating a synergistic effect between the two compounds. *Conclusions*: This study is the first to demonstrate that Hes induces apoptosis in U2OS osteosarcoma cells and that its combination with Cis may enhance anticancer efficacy by activating apoptosis-related cell death pathways. Given the growing focus on combination therapies and cell death mechanisms in cancer research, these findings provide valuable insights into potential novel strategies for osteosarcoma treatment.

## 1. Introduction

Osteosarcoma is a malignant tumor originating from bone tissue, most commonly affecting the knee and shoulder regions. It represents the most prevalent primary bone malignancy in children and adolescents. The standard treatment for osteosarcoma involves a combination of chemotherapy and surgical resection, with early diagnosis being a critical determinant of treatment success [1]. Despite being the most common primary bone cancer in the adolescent population, osteosarcoma is associated with a poor prognosis, particularly after metastasis. Although considerable progress has been made in understanding the disease, the five-year survival rate has improved only marginally, underscoring the limitations of current therapeutic approaches [2,3].

Cis and doxorubicin are among the most frequently used chemotherapeutic agents, either as monotherapy or in combination, for the treatment of osteosarcoma. Cis functions primarily by inducing DNA damage in tumor cells, thereby inhibiting cell proliferation and triggering apoptosis. However, a major challenge associated with Cis therapy is the emergence of drug resistance in some patients. This resistance reduces treatment efficacy, extends the duration of therapy, and compromises clinical outcomes [4]. In light of these limitations, the development of novel therapeutic strategies, including combination treatments, is gaining increasing attention to overcome Cis resistance.

Over the past four decades, natural compounds have played a pivotal role in the discovery and development of anticancer agents. Bioactive molecules derived from natural sources, as well as their synthetic or semi-synthetic derivatives, have served as essential lead compounds in the design of more effective and targeted cancer therapies. Numerous studies have demonstrated that flavonoids play a significant role in cancer chemotherapy. These compounds interact with a wide range of genes and enzymes, modulating various molecular pathways involved in tumor progression. Their mechanisms of action include induction of cell cycle arrest, promotion of apoptosis, and inactivation of carcinogens [5]. Hesperidin, a naturally occurring bioflavonoid belonging to the coumarin family, has garnered increasing attention for its anticancer potential. Coumarins are well-documented for their diverse biological activities, including anticancer effects. They have been reported to act as immunomodulators in malignant melanoma [6], to exert antiproliferative effects in bladder cancer [7], and to induce apoptosis in MG-67 osteosarcoma cells both in vitro and in vivo [8]. For example, curcumol, a bioactive compound, has been shown to enhance the cytotoxic effects of Cis when used in combination therapy, significantly suppressing osteosarcoma tumor growth. Moreover, recent studies have identified the enzyme Otulin as a critical factor in the development of Cis resistance. Targeting Otulin may represent a promising strategy to overcome chemoresistance and improve therapeutic outcomes in osteosarcoma treatment [9,10].

Members of the Bcl-2 protein family are essential regulators of apoptotic cell death. Aberrant overexpression of pro-survival Bcl-2 family proteins or reduced expression of pro-apoptotic Bcl-2 family proteins, both of which suppress apoptosis, are frequently observed in various types of cancer. Due to their central role in the regulation of apoptosis, these proteins are considered promising targets for the development of new anticancer therapies [11]. Bax, a pro-apoptotic protein within the Bcl-2 family, plays a critical role in the initiation of apoptosis. When Bax expression is dominant, it promotes apoptotic activity. In response to apoptotic signals, the synthesis of Bax increases, which neutralizes the anti-apoptotic effects of Bcl-2 and enhances the apoptotic response. Under normal physiological conditions, Bax is localized in the cytosol. However, during apoptosis, it translocates to the mitochondrial membrane, where it contributes to the formation of pores, leading to the release of cytochrome c and the activation of downstream apoptotic pathways. The loss of Bax function has been associated with the development of certain leukemias and colorectal cancers [12].

In this study, we demonstrated that the combination of Hes and Cis exerts synergistic antitumor effects on osteosarcoma cells, as evidenced by cell proliferation, apoptosis, and migration assays. Specifically, our findings indicate that this combination enhances the sensitivity of osteosarcoma cells to Cis, suggesting its potential as a promising therapeutic strategy for osteosarcoma patients. Although numerous studies have reported the potent anticancer effects of phytochemicals with alternative therapeutic properties, biological challenges such as the selective targeting of cancer cells, efficient cellular delivery, and initiation of specific molecular interactions remain significant obstacles. These limitations highlight the importance of developing combination therapies, particularly those involving two or more agents, to achieve improved therapeutic outcomes. Currently, increasing attention is being directed toward combination treatments and the modulation of cell death pathways in cancer research. To our knowledge, this is the first study to demonstrate the therapeutic potential of a combined Hes and Cis treatment in osteosarcoma, providing novel insights into their synergistic mechanisms of action.

## 2. Material and Methods

### 2.1. Chemicals

Hesperidin (Sigma-Aldrich, H5254, ≥80%, 25 g), MTT, and RPMI-1640 medium were purchased from Sigma-Aldrich (Hamburg, Germany). Cisplatin (50 mg/100 mL, intravenous formulation) was obtained from Orna Farma (Koçak Pharma, Istanbul, Türkiye). Fetal bovine serum (FBS), penicillin, and streptomycin were purchased from Thermo Fisher Scientific (Waltham, MA, USA).

### 2.2. Cell Culture

The human osteosarcoma cell line U2OS was obtained from Cytion (Catalog No. 300364, Eppelheim, Germany). Following verification, the cells were expanded for stock, cryopreserved, and cultured in 10% dimethyl sulfoxide (DMSO) (Sigma-Aldrich, St. Louis, MO, USA). Cell lines were passaged every three days and maintained for a maximum duration of two months. Short Tandem Repeat (STR) analysis was performed to authenticate the identity of all cell lines. Cells were maintained in RPMI-1640 medium supplemented with 10% fetal bovine serum (FBS) and incubated at 37 °C in a humidified atmosphere containing 5% CO_2_.

### 2.3. MTS Assay

To evaluate cytotoxicity in cells treated with Hes and Cis, the CellTiter 96^®^ AQueous One Solution Cell Proliferation Assay (MTS) was obtained from Promega Corporation, Madison, WI, USA. This assay utilizes the reagent 3-(4,5-dimethylthiazol-2-yl)-5-(3-carboxymethoxyphenyl)-2-(4-sulfophenyl)-2H-tetrazolium to measure cell viability. A total of 5 × 10^3^ human osteosarcoma U2OS cells were seeded into 96-well plates, incubated overnight, and subsequently treated with increasing concentrations of Hes (0, 5, 25, 50, 100, 150, 200, and 250 µM) and Cis (0, 10, 20, 30, 40, 50, 60, and 70 µM). Following 48 h of incubation, the MTS reagent was added to each well in accordance with the manufacturer’s protocol. Absorbance was measured at 492 nm using a Multiskan GO microplate reader (Thermo Scientific, Waltham, MA, USA).

### 2.4. Determination of IC_50_

Based on the results of the MTS assay, the IC_50_ values for Hes and Cis in both control and experimental groups were determined using Combenefit v2.021 software to assess the effectiveness of each compound at varying concentrations.

### 2.5. Antagonist–Synergist Effect

To evaluate the antagonistic or synergistic interaction between Hes and Cis, eight different concentrations of each compound were prepared using serial dilution, resulting in 64 distinct combination conditions. Following 48 h of incubation, cell viability was assessed using the MTS assay. The dose–response curves for each compound were established in 96-well plates with five replicates per condition. The data obtained from the MTS assay were analyzed using Combenefit software (Cambridge, UK) to determine potential synergistic or antagonistic effects between the two agents. After identifying the IC_50_ values, experimental groups were established using the determined IC_50_ doses for U2OS cells. The IC_50_ values were calculated through probit analysis using the SPSS version 20 statistical software package. The resulting IC_50_ doses were subsequently used in all further experimental procedures.

In addition to the Chou–Talalay CI and HSA models, the Bliss Independence and Loewe Additivity models were also applied using Combenefit software to validate the observed interaction between Hes and Cis. These models offer complementary perspectives on drug synergy by evaluating expected versus observed effects. The inclusion of multiple interaction models enhances the methodological robustness of our findings and addresses variability in synergy interpretation across different frameworks.

### 2.6. Hoechst 33342 Fluorescent Staining

The cell death induced by Hes and Cis in U2OS cells was assessed using Hoechst 33342 (Thermo Scientific, Waltham, MA, USA), a fluorescent dye that specifically stains nuclei and highlights apoptotic features through morphological changes such as chromatin condensation and nuclear fragmentation. Osteosarcoma cells were seeded in 24-well plates at a density of 5 × 10^4^ cells per well and incubated under standard conditions. After 24 h, cells were treated with Hes IC_50_, Cis IC_50_, and Hes IC_50_ + Cis IC_50_ concentrations, based on the 48 h IC_50_ values previously determined. Live-cell staining was performed in accordance with the manufacturer’s protocol, and cells were incubated at 37 °C for 30 min. Fluorescent images were then acquired using a DAPI filter at 20× objective magnification with the EVOS^®^ FL Imaging System was obtained from Thermo Fisher Scientific, Waltham, MA, USA.

### 2.7. Caspase-3/7 Activity Assay

The Caspase-3/7 Glo Assay kit from Promega (Madison, WI, USA) was used to confirm the activity in the apoptosis pathway. Cells were treated in 96-well plates; caspase reagent was added, according to the kit procedure, at the end of the incubation and the cells were then left in the dark for 1 h at room temperature. Luminescence values were recorded with the assistance of a microplate reader and compared to the control group.

### 2.8. Inflammatory Cytokines (TNF-α, IFN-γ, IL-1, IL-6)

To evaluate the anti-inflammatory effects of crocin on MCF-7 cells, the levels of TNF-α (Invitrogen, Cat. No. 13-7341-81), IL-1β (Invitrogen, Cat. No. PA1-84913), IL-6 (Cat. No. EH2IL6), and IFN-γ (Invitrogen, Waltham, MA, USA, Cat. No. 14-7311-81) were measured spectrophotometrically at 540 nm.

### 2.9. Gene Expression

Quantitative gene expression analysis was conducted to evaluate the effects of Hes and Cis on pro-apoptotic and anti-apoptotic gene expression in U2OS cells. For this purpose, specific primer sets targeting the genes of interest were designed. The β-actin gene was used as an internal reference to normalize gene expression levels. Relative changes in gene expression were calculated using the threshold cycle (Ct) values.

### 2.10. Total RNA Isolation

For total RNA isolation, osteosarcoma cells were seeded into 6-well plates and treated with Hes IC_50_, Cis IC_50_, and the Hes IC_50_ + Cis IC_50_ combination. Following treatment, the cells were harvested by adding 1 mL of cold phosphate-buffered saline (PBS) to each well. Total RNA was isolated using a commercial kit, in accordance with the manufacturer’s instructions (Thermo Fisher Scientific, Waltham, MA, USA). The purity and concentration of the RNA were assessed using an Optizen NanoQ micro-volume spectrophotometer (Mecasys, Yuseong-gu, Republic of Korea). RNA samples were then diluted with ultrapure water to a final concentration of 750 ng/10 μL. Subsequently, complementary DNA (cDNA) synthesis was performed using the corresponding kit, following the manufacturer’s protocol (Bio-Rad, Hercules, CA, USA). The synthesized cDNA samples were stored at –20 °C, while the remaining RNA samples were preserved at –86 °C for further analyses.

### 2.11. RT-qPCR

Complementary DNAs (cDNAs) were amplified using the SYBR Green-based quantitative PCR kit (2× qPCRBIO SyGreen Mix Lo-ROX Kit, PCR Biosystems, London, UK). The expression levels of Bax, Bcl-2, Caspase-3, and Survivin—key genes involved in the apoptotic pathway—were assessed in control and treatment groups of osteosarcoma (U2OS) cells using the real-time quantitative PCR (RT-qPCR) method. β-Actin was employed as the reference housekeeping gene for normalization. Gene amplification and quantification were performed using a Bio-Rad CFX96 real-time PCR system. The untreated group served as the negative control. The forward and reverse primer sequences used in the RT-qPCR are listed in Table 1. The qPCR cycling conditions were as follows: initial denaturation at 95 °C for 2 min, followed by 40 cycles at 95 °C for 5 s, 45 cycles at 66 °C for 45 s, 45 cycles at 74 °C for 2 min, and a final extension step at 72 °C for 5 min. Relative mRNA expression levels were calculated using the 2^−ΔΔCt^ method. All cDNA and standard samples were analyzed under identical conditions and in triplicate to minimize intra-group variability. Expression of endogenous β-actin mRNA was used as an internal control for both calibration and normalization.

### 2.12. Statistical Analysis

The differences in cell viability ratios, as determined by the MTS assay, and the mean gene expression levels obtained from RT-qPCR analyses were evaluated using two-way analysis of variance (ANOVA). Post hoc comparisons were conducted using the Tukey Honestly Significant Difference (HSD) test to identify statistically significant differences between groups. For pairwise comparisons, the independent samples *t*-test was applied, provided that the assumption of data homogeneity was met. All statistical analyses were performed using SPSS version 20 (IBM, Armonk, NY, USA), with a *p*-value of <0.05 considered statistically significant.

## 3. Results

### 3.1. Cytotoxicity

When osteosarcoma (U2OS) cells cultured in flasks reached 90% confluency, they were seeded into 96-well plates at a density of 5 × 10^3^ cells per well. Hes and Cis were applied individually and in combination. At the end of the treatment period, cell viability was assessed using the MTS assay. The optical density (OD) value of the untreated control group was considered to represent 100% cell viability. The cell viability in each treatment group was calculated using the following formula:

% Viability = (OD of test group × 100)/OD of control group

Hes exhibited strong cytotoxic activity in human osteosarcoma U2OS cells. The results demonstrated that the cytotoxic effects of Hes increased in a dose-dependent manner within the concentration range of 0–250 µM. A significant enhancement in cytotoxicity was observed when the dose of Hes was increased from 50 µM to 100 µM. To quantitatively assess the cytotoxic potency of Hes, the 48 h IC_50_ value was calculated and found to be 106 µM in U2OS cells.

**Figure 1:**
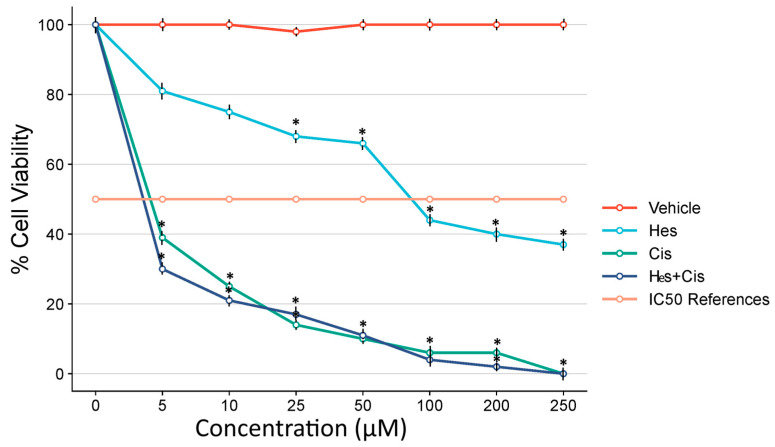
Dose–response curves of Hes and Cis in U2OS osteosarcoma cells. The graph illustrates the inhibitory effects of increasing concentrations of Hes (0–250 µM, yellow line with circles) and Cis (0–70 µM, orange line with squares) on U2OS cell viability after 48 h of treatment. Cell viability was assessed using the MTS assay, and the results are expressed as a percentage relative to the untreated control group (100% viability). The dashed horizontal line indicates the 50% viability threshold (IC_50_ reference). A dose-dependent decrease in cell viability was observed for both compounds. The IC_50_ value for Hes was determined to be 106 µM, whereas Cis exhibited a much stronger cytotoxic profile, with an IC_50_ of 4.83 µM. All results are presented as mean ± standard deviation (SD) from at least five independent experiments. Statistical analyses were performed using two-way ANOVA followed by Tukey’s post hoc test for multiple group comparisons. A *p*-value < 0.05 was considered statistically significant. The difference in cell viability at IC_50_ concentrations of both Hes and Cis, as well as when they were combined, was found to be statistically significant when compared to the control group (* *p* < 0.001). These results indicate a synergistic cytotoxic effect, especially at higher combination doses. (The horizontal dashed line marks 50% cell viability, serving as a visual reference for determining the IC_50_ value of the drug).

Cis, a well-established chemotherapeutic agent used in the treatment of various cancers including osteosarcoma, demonstrated an IC_50_ value of 4.83 µM after 48 h of treatment. A statistically significant reduction in cell proliferation was observed with increasing concentrations of both Hes and Cis. Statistical analysis indicated that cell viability was significantly reduced, particularly at Hes and Cis concentrations exceeding their respective IC_50_ values, compared to the vehicle-treated control group (Figure 1).

When comparing Hes and Cis treatments, a greater reduction in U2OS cell viability was observed in the Cis-treated group, and this difference was statistically significant. Both Hes and Cis exhibited marked cytotoxic effects once their respective IC_50_ concentrations were reached. A statistically significant difference in cell viability was also observed when compared to the vehicle-treated control group (*p* < 0.05).

These findings confirm that Hes and Cis both exhibit cytotoxic effects on U2OS cells, with Cis being more potent. However, the heatmap suggests that Hes enhances the cytotoxicity of Cis when the agents are combined, supporting its potential as a chemosensitizer in osteosarcoma therapy.

### 3.2. Antagonism/Synergism

**Figure 2:**
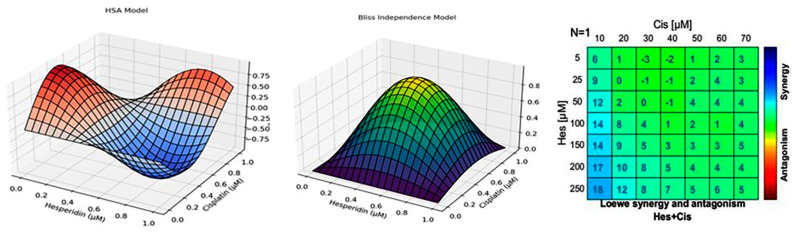
**Left Panel (HSA Model):** The 3D surface plot illustrates synergy scores derived from the Highest Single Agent (HSA) model. The X and Y axes represent increasing concentrations of Hes and Cis, respectively, while the *Z*-axis shows the synergy score. Blue regions indicate synergy (positive interaction), red regions indicate antagonism (negative interaction), and neutral (white/light) regions represent additive effects. **Middle Panel (Bliss Independence Model):** This 3D plot represents the Bliss Independence model, which assesses whether the combined effect of Hes and Cis exceeds the expected effect, assuming independent action. Similar to the HSA model, synergy is indicated by positive peaks, antagonism by valleys, and flat areas no effect. **Right Panel (Loewe Additivity Heatmap):** A heatmap based on the Loewe Additivity model shows synergy and antagonism scores for various Hes–Cis combinations. Each cell is color-coded, with blue indicating high synergy and red indicating antagonism. The numeric values in the cells denote the synergy scores. The heatmap clearly demonstrates a dose-dependent increase in synergy, especially at higher Hes concentrations (200–250 µM) combined with moderate Cis doses (10–40 µM).

In this study, Hes and Cis treatments demonstrated dose-dependent inhibition of proliferation in the U2OS cell line. In combined applications, Hes and Cis reduced the Cis dose required to achieve cytotoxicity in osteosarcoma cells. This synergistic effect was significant and observed across all combined doses of Hes and Cis (Figure 2).

Notably, Hes and Cis exhibited a synergistic effect according to HSA scores, even when applied in combination at doses below their respective IC_50_ values. This effect was also statistically significant. These results indicate that the combined application of Hes and Cis produced a strong synergistic effect in the U2OS cell line (Figure 2). Based on these findings, subsequent analyses in this study were conducted using combined doses of Hes and Cis.

To further evaluate the reliability of the observed synergistic interaction, additional modeling was performed using the Bliss Independence and Loewe Additivity frameworks (Figure 2). The Bliss model demonstrated moderate to strong synergy across several mid-range concentration combinations, while the Loewe model highlighted pronounced synergistic interactions at higher Hes and Cis doses. These consistent findings across different models confirm that Hes enhances the antitumor efficacy of Cis in a dose-dependent manner. When the Cis treatment dose was taken as 10 µM and the Hes treatment was applied in the dose range of 50–250 µM, strong synergy emerged in the Loewe model. This situation is quite positive for cancer patients who develop Cis resistance, because it has been shown that Hes can be used as a synergistic agent for Cis, whose toxic effects sometimes reach unbearable levels at high doses. However, it is not possible to make direct clinical dosage recommendations based on these findings. For this purpose, combination trials with variable ratios in addition to fixed ratios in wider dose ranges have been suggested (Figure 2).

The synergy data reinforce findings from the Chou–Talalay and Bliss analyses, confirming a robust interaction across multiple modeling frameworks. Experimental data were obtained from triplicate assays and analyzed via Combenefit [13].

To quantitatively validate the synergistic interaction between Hes and Cis observed in U2OS cells, the Chou–Talalay Combination Index (CI) model was applied. This model is widely recognized for its ability to rigorously assess drug–drug interactions in combination therapies. The CI value was calculated using the following formula:

CI = (D_1_/D_x1_) + (D_2_/D_x2_)

where:

- D_1_ and D_2_ represent the doses of Hes and Cis used in combination;
- D_x1_ and D_x2_ refer to the doses required to achieve the same inhibitory effect (e.g., 50%) when each drug is used individually. These findings are in agreement with the dose–response shift observed in the combination treatment groups and further support the conclusion that Hes enhances the cytotoxic efficacy of Cis in osteosarcoma cells.

The Combination Index (CI) values were calculated to evaluate the interaction between Hes and Cis in U2OS osteosarcoma cells. A CI value < 1 indicates synergism, a CI value = 1 indicates an additive effect, and a CI value > 1 indicates antagonism. According to the Chou–Talalay, Bliss Independence, and Loewe Additivity models, the combination of Hes and Cis consistently exhibited synergistic effects, with CI values ranging from 0.58 to 0.65. These findings support the enhanced cytotoxic and pro-apoptotic effects observed in the combination treatment compared to individual treatments (Table 2).

### 3.3. Caspase-3/7 Activity

**Figure 3:**
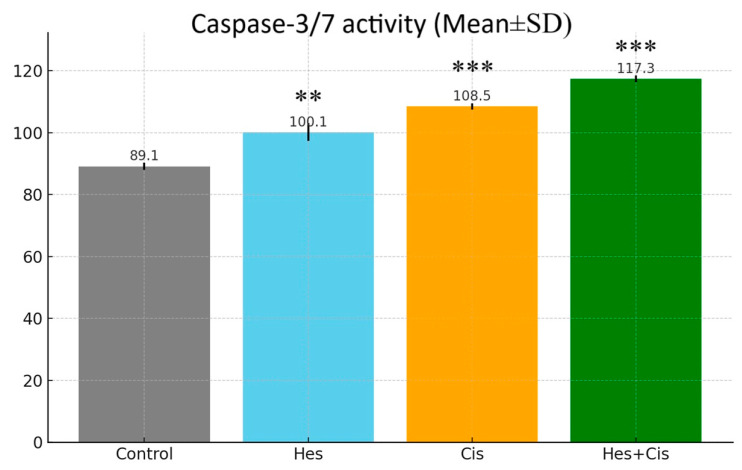
Caspase 3/7 activity measured in Control, Hes-, Cis-, and Hes + Cis-treated cells. Bars represent mean ± SD (*n* = 3). Statistical analysis was performed using two-way ANOVA followed by Tukey’s post hoc test. Asterisks (** and ***) indicate statistically significant differences between groups (** *p* < 0.01, *** *p* < 0.001).

Hes and Cis treatments directly affected caspase 3/7 activity. Apoptosis occurs in cells with the activation of caspase proteases. In our study, we investigated caspase activity in the groups where Hes, Cis, and Hes + Cis were applied to measure apoptotic cell death. A significant increase in caspase activity in the Hes and Cis groups was reflected in the statistical data. However, the biggest difference in caspase activity was detected in the Hes + Cis group (*p* < 0.001) (Figure 3). In addition, when the groups in which Hes and Cis were applied separately were compared with the Hes + Cis group, a statistically significant difference occurred (*p* < 0.001 (for Hes), *p* < 0.003 (for Cis)). These results were parallel to the results of the changes in the dead and live cell ratios measured by Hoechst 33342 staining.

### 3.4. Inflammatory Cytokine Levels

**Figure 4:**
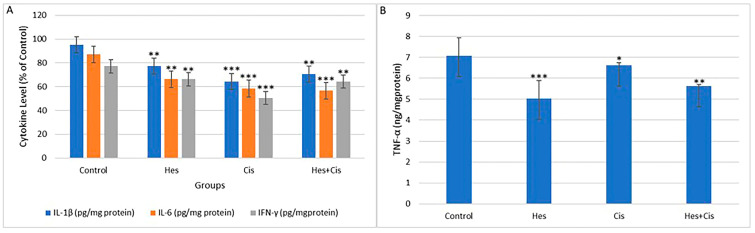
Effects of Hes and Cis on inflammatory cytokine levels in U2OS osteosarcoma cells. Bar chart illustrating the relative expression levels of key pro-inflammatory cytokines—IL-1β, IL-6, TNF-α, and IFN-γ—across four experimental groups: untreated control, Hes-treated, Cis-treated, and Hes + Cis combination-treated (**A**,**B**). Cytokine levels were normalized to the control group and expressed as a percentage (% of control). A significant reduction in cytokine expression was observed in all treatment groups compared to the control. The most pronounced decrease in IL-6 and IFN-γ levels occurred in the Hes + Cis combination group, while TNF-α and IL-1β also showed marked suppression, particularly in the individual Hes and Cis treatment groups. These findings suggest that both agents exert anti-inflammatory effects on osteosarcoma cells, with enhanced potency observed under combination treatment. Data represent the average of three independent experiments (*n* = 3). Statistical significance was evaluated using two-way ANOVA with Tukey’s post hoc test (* *p* < 0.05, ** *p* < 0.01, *** *p* < 0.001).

In U2OS cells, changes in the levels of TNF-α, IL-1, IL-6, and IFN-γ, which are indicators of inflammation, were found to be statistically significant compared to the control group (Figure 4A,B). The greatest reduction in IL-1 and IL-6 levels was observed in the Cis group, while the most significant effect on inflammation was seen in the Hes group compared to the control group.

The reduction in TNF-α and IL-1β levels was statistically significant between the control group and the Hes + Cis treatment group (*p* < 0.01) (Figure 4A,B). Additionally, the decrease in IFN-γ levels was also found to be statistically significant (*p* < 0.01) (Figure 4A).

Overall, the results suggest that Hes and Cis, both individually and in combination, significantly reduce the levels of inflammation-related cytokines in U2OS cells. The combination treatment demonstrated the most pronounced effect, supporting its potential as a synergistic anti-inflammatory therapeutic strategy.

### 3.5. Nuclear Condensation and Fragmentation Results

**Figure 5:**
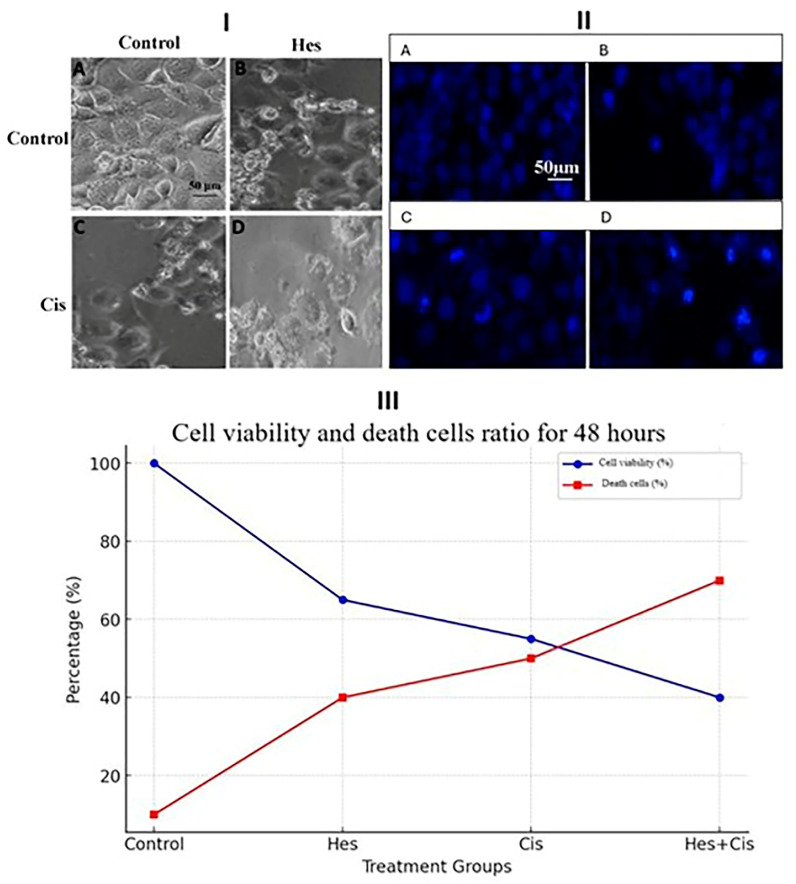
(**I**) Morphological changes, Hoechst 33342 staining, and quantitative analysis of cell viability and cell death in osteosarcoma cells after 48 h of treatment. Representative phase-contrast images show cellular morphology in the control, Hes, Cis, and Hes + Cis treatment groups. (**II**) Hoechst staining (panels A–D) demonstrates nuclear condensation and fragmentation indicative of cell death, with the most intense fluorescence observed in the combination group. (**III**) The graph illustrates the percentage of viable and dead cells across treatment groups. All data were analyzed using two-way ANOVA followed by Tukey’s post hoc test for multiple group comparisons. Each condition was tested in triplicate (*n* = 3), and values are expressed as mean ± standard deviation (SD). A *p*-value of <0.05 was considered statistically significant. These findings further support that Hes enhances Cis-induced cell death in U2OS cells.

To confirm the apoptotic cell death indicated by the MTS assay, Hoechst 33342 staining was performed on Hes- and Cis-treated U2OS cells. The results demonstrated a dose-dependent increase in cell death in both treatment groups (Figure 5(I)). The observed nuclear fragmentation was indicative of apoptosis-associated cytotoxicity. Cells with nuclear condensation or fragmentation observed as a result of staining with Hoechst 33342 were evaluated as apoptosis-associated nuclear changes (Figure 5(II)). However, Hoechst 33342 staining alone does not confirm apoptosis and was used here to support morphological observations, which were further validated with caspase-3/7 activity assays and quantified via live/dead cell analysis, as shown in Figure 5(III).

### 3.6. Gene Expression Findings

**Figure 6:**
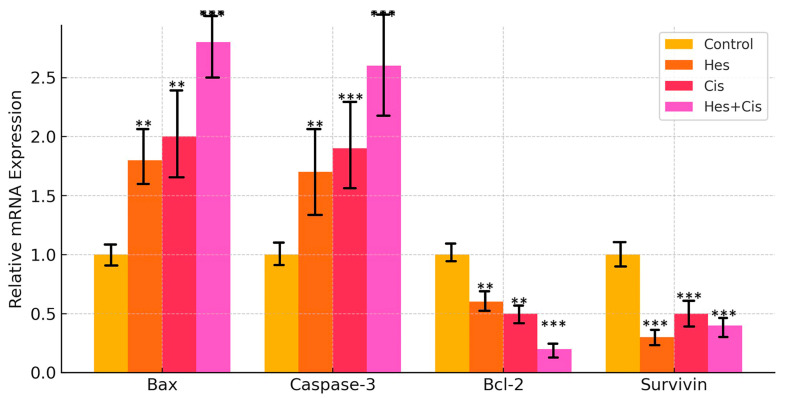
Relative mRNA expression levels of apoptosis-related genes (Bax, Caspase-3, Bcl-2, and Survivin) in different treatment groups (Control, Hes, Cis, and Hes + Cis) after 48 h. Gene expression was quantified using RT-qPCR and normalized to β-actin as the internal control using the 2^−ΔΔCt^ method. Data are presented as mean ± standard deviation (SD) from three independent experiments (*n* = 3). The pro-apoptotic markers Bax and Caspase-3 were significantly upregulated in all treatment groups compared to control, with the highest expression levels observed in the combination group. Conversely, the anti-apoptotic genes Bcl-2 and Survivin were significantly downregulated, particularly in the combination treatment, indicating activation of the intrinsic mitochondrial apoptotic pathway. Statistical analysis was performed using two-way ANOVA followed by Tukey’s post hoc test (** *p* < 0.01, *** *p* < 0.001).

The expression levels of apoptosis-related genes (Bax, Bcl-2, Caspase-3, and Survivin) in osteosarcoma (U2OS) cells were analyzed by comparing the Hes- and Cis-treated groups to the control group. Treatment with Hes and Cis at their respective IC50 concentrations significantly upregulated the expression of Bax and Caspase-3 genes compared to the control group (** *p* < 0.01). In the combination treatment group (Hes + Cis at IC50), Bax and Caspase-3 expression levels reached their highest values in U2OS cells (*** *p* < 0.001) (Figure 6).

Conversely, expression of the anti-apoptotic genes Bcl-2 and Survivin was significantly downregulated in both Hes- and Cis-treated cells compared to the control group (** *p* < 0.01) (Figure 6). Notably, Bcl-2 expression showed the greatest decrease in the combined Hes + Cis treatment group (*** *p* < 0.001), while Survivin expression was most significantly reduced following Hes treatment alone (*** *p* < 0.001) (Figure 6).

### 3.7. Gene Ontology

**Figure 7:**
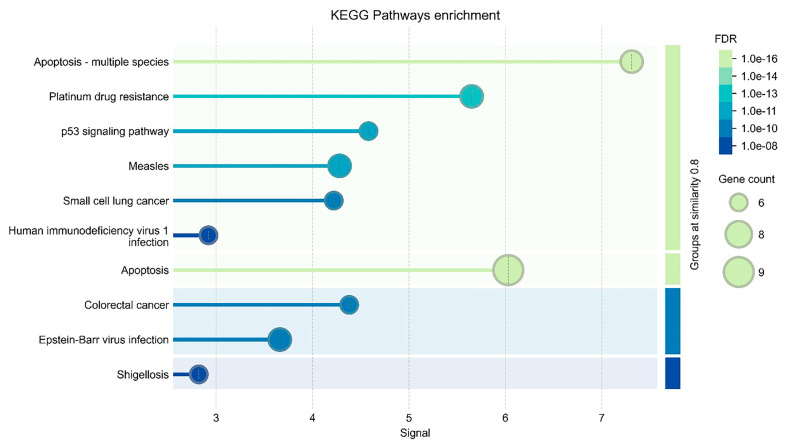
This image represents a functional enrichment analysis visualization, likely obtained from a STRING database analysis or a similar bioinformatics tool. It highlights the involvement of specific genes in various biological processes, cellular components, or molecular functions. The FDR (False Discovery Rate) is displayed in a color gradient from light green, representing the most statistically significant pathways (FDR < 1.0 × 10^−16^), to dark blue, indicating pathways with lower significance (FDR~1.0 × 10^−8^). The gene count is represented by bubble size, with larger circles indicating a higher number of genes associated with a particular pathway or function.

In the BP (biological process) category of target genes, Bax signaling is primarily involved in apoptotic signaling (Figure 7). Regarding cellular components, Bax initiates apoptosis through cytochrome c release from mitochondria. In the MF (molecular function) category, its role in BH binding protein interactions is prominent. In the CC (cellular component) category, its association with Bcl-2 family proteins is highlighted (Figure 7).

BP, MF, and CC pathway enrichment analyses of target genes were performed using the STRING program. The findings revealed that 502 genes were involved in the enrichment process, with 122 pathways associated with apoptosis showing statistical significance (*p* < 0.05) (Figure 7).

The pathways and connections are visualized with horizontal lines and circles, showing the relationship between gene clusters and their functional categories. This analysis suggests that multiple gene sets are significantly enriched in various biological functions, with some having very high statistical significance. This visualization helps identify the most relevant pathways involved in the analyzed dataset that are potentially related to apoptosis, cellular signaling, or disease mechanisms.

## 4. Discussion

In the clinical management of osteosarcoma, Cis-based chemotherapy remains a cornerstone of treatment; however, its efficacy is often limited by the development of drug resistance and adverse side effects. Consequently, the identification of novel therapeutic strategies, including combination therapies, is essential to improve treatment outcomes. In this study, we demonstrated that Hes and Cis exert a synergistic effect on U2OS osteosarcoma cells by enhancing apoptosis through the mitochondrial pathway. Our results showed that both Hes and Cis significantly upregulated the expression of the pro-apoptotic genes Bax and Caspase-3, while downregulating the anti-apoptotic genes Bcl-2 and Survivin, indicating the activation of apoptotic mechanisms. Moreover, the combination treatment resulted in a more pronounced reduction in cell proliferation compared to either agent alone, suggesting that Hes may sensitize osteosarcoma cells to Cis-induced apoptosis. While the Chou–Talalay model is a standard method for assessing combination indices, the use of the Bliss and Loewe models in this study provides additional layers of validation. Their integration reinforces the conclusion that the Hes–Cis combination exerts a reproducible synergistic effect that is not limited to a single modeling approach. This methodological diversity strengthens the translational potential of our findings. Furthermore, the calculated CI values (Table 2) corroborated the observed synergistic interactions across multiple analytical models, confirming the robustness and reproducibility of the combination effect between Hes and Cis.

These findings underscore the potential of Hes as a chemosensitizer in osteosarcoma therapy and support further investigation into its clinical applicability. In clinical practice, Cis-based therapy remains a cornerstone in the treatment of osteosarcoma. Despite numerous combination therapies being evaluated over the past four decades, the long-term survival rate of osteosarcoma patients has shown little improvement [14,15]. This highlights a critical bottleneck in current chemotherapy approaches that must be addressed [16]. In this study, we propose a novel and effective combination therapy for osteosarcoma. Hes was found to sensitize osteosarcoma cells to Cis-induced proliferation suppression in a time- and dose-dependent manner over a 48 h period. Furthermore, the combined treatment of Hes and Cis significantly induced apoptosis. Our findings demonstrate that Hes exerts a strong synergistic effect when used in combination with Cis. These results offer promising implications for improving therapeutic strategies in osteosarcoma patients.

In this study, the cytotoxic effect of Hes on apoptosis in U2OS osteosarcoma cells was demonstrated through its combined application with Cis. The apoptosis induced by Hes and Cis was activated via stimulation of Bax, Bcl-2, and Caspase-3, thereby initiating the intrinsic apoptotic pathway through mitochondrial activation. While Hes and Cis upregulated the expression of pro-apoptotic proteins (Bax and Caspase-3), they concurrently downregulated the expression of anti-apoptotic proteins (Bcl-2 and Survivin). These proteins are known to regulate intracellular apoptotic pathways and may enhance the sensitivity of U2OS cells to treatment. Notably, the findings from this study are consistent with those reported in studies using the MG-63 osteosarcoma cell line [8]. A significant increase in the expression of both early and late apoptotic markers was observed following single and combined treatments with Hes and Cis (*p* < 0.001), further supporting the potential of this combination in inducing apoptosis in osteosarcoma cells.

In recent years, combining natural compounds with conventional therapeutic agents has attracted considerable attention, not only in cancer research but also in studies involving various disease models [17,18]. The incorporation of natural agents into combination therapies for cancer has been shown to exert chemoprotective effects against anticancer drugs while simultaneously enhancing their anti-proliferative activity [8,19,20]. A review of the literature revealed that Hes has previously been applied to the MG-63 osteosarcoma cell line; however, no study to date has investigated the effects of Hes alone or in combination with Hes + Cis on U2OS cells [21]. This study is the first to demonstrate the anti-proliferative effects of the Hes + Cis combination in U2OS osteosarcoma cells.

In this study, single doses of Cis and Hes were initially administered and their IC50 values determined. Subsequently, the combination index was calculated, and various combination doses were applied to evaluate their effects on cell viability. Consequently, a synergistic effect was observed with the combination of 106 μM Hes and 4.83 μM Cis.

The present study demonstrated that oxidative stress was elevated in the group treated with Hes and Cis, likely due to a reduction in inflammatory cytokine levels. As a result, the expression of Bax, a pro-apoptotic gene, was upregulated. Numerous studies have reported that oxidative stress mediators originating from the cytosol and mitochondria induce apoptosis by activating apoptotic genes such as p53 [22]. Furthermore, the combination treatment triggered apoptosis through the activation of Caspase-3. In certain cases, apoptosis may also be initiated in response to misfolded proteins, a process associated with endoplasmic reticulum (ER) stress. However, further studies are required to elucidate this mechanism. It is crucial to investigate agents that enhance PARP cleavage following the activation of effector caspases such as Caspase-3, -6, and -7. Some studies have demonstrated that curcumin can induce PARP cleavage [23].

Previous studies have reported that coumarins exhibit immunomodulatory activity against malignant melanoma [6]. In a study on bladder cancer, labeled coumarins were shown to suppress cell proliferation, highlighting their potential utility in clinical applications [7]. Notably, this activity was observed even in the absence of UV radiation. Coumarins have also been found to affect the adhesion and motility of certain neoplastic cells; moreover, their impact on an invasive mouse melanoma cell line has been described. The processes of proliferation and survival signaling in cancer cells involve complex interactions among growth factors such as epidermal growth factor (EGF), transforming growth factor (TGF)-α, and TGF-β [24]. Studies conducted on various cell lines have shown that Hes exerts anticarcinogenic effects by inhibiting cell proliferation. In the present study, it was observed that Hes, when combined with Cis, significantly downregulated the mRNA expression of Bcl-2, a key gene associated with cellular proliferation. These findings are in agreement with those of previous studies [25,26,27]. In the treatment of cancers such as osteosarcoma, chemotherapy and radiotherapy remain indispensable. Therefore, the development of novel therapeutic strategies is of great importance [28].

While the current study provides robust in vitro evidence for the synergistic antitumor effects of Hes and Cis in osteosarcoma cells, several important limitations must be acknowledged. First, the experiments were conducted in conventional 2D monolayer cultures, which do not replicate the complex three-dimensional architecture of the tumor microenvironment. Recent studies have demonstrated that tumor progression is strongly influenced by the biophysical properties of the ECM, including its stiffness and spatial organization, which modulate cellular behaviors through mechanotransduction and integrin signaling [29]. These properties are not adequately modeled in 2D cultures, which may limit the translational relevance of the observed drug responses. Second, the tumor ECM, often referred to as the oncomatrix, undergoes dynamic remodeling during cancer development and metastasis, influencing cell adhesion, signaling, and drug sensitivity. The evolving biochemical and mechanical landscape of the oncomatrix plays a crucial role in tumor heterogeneity and resistance mechanisms but was not addressed in this study [30]. Third, although multiple drug synergy models including the Chou–Talalay, Bliss, Loewe, and HSA models were applied to validate the interaction between Hes and Cis, these computational frameworks do not incorporate the physiological feedback loops, ECM interactions, or pharmacokinetic dynamics that are present in vivo. Future studies using 3D tumor spheroids, biomimetic hydrogels, or animal models are needed to confirm and extend these findings under more physiologically relevant conditions.

Future perspectives for the application of Hes in osteosarcoma treatment are promising yet require further exploration. Based on the synergistic antitumor effects observed in this study, future research should prioritize validating the efficacy and safety of Hes and Cis combination therapy in appropriate in vivo osteosarcoma models. Additionally, it will be important to investigate the pharmacokinetics, optimal dosing schedules, and potential toxicity profiles of this combination to ensure its clinical applicability. Molecular studies are also needed to clarify the upstream regulators and downstream signaling pathways beyond Bax and Bcl-2 modulation, including the roles of mitochondrial integrity, oxidative stress responses, and endoplasmic reticulum stress mechanisms. Furthermore, assessing the effects of Hes in combination with other chemotherapeutic agents could expand its therapeutic potential. Finally, the development of advanced drug delivery systems such as nanoparticle-based formulations may improve the bioavailability, stability, and tumor-targeting efficiency of Hes, supporting its transition from experimental research to clinical application in osteosarcoma management.

## 5. Conclusions

This study is the first to demonstrate that the combination of Hes and Cis exerts a synergistic antitumor effect in human osteosarcoma U2OS cells by inducing mitochondrial-mediated apoptosis. The combined treatment significantly increased the expression of pro-apoptotic genes such as Bax and Caspase-3, while reducing the levels of anti-apoptotic markers including Bcl-2 and Survivin. These molecular changes support the activation of the intrinsic apoptotic pathway. Furthermore, the treatment was associated with a notable decrease in inflammatory cytokine levels, suggesting an additional anti-inflammatory effect.

The results highlight the potential of Hes as a promising chemosensitizing agent that can enhance the therapeutic efficacy of Cis. This combination may also contribute to reducing the required dose of Cis, thereby minimizing its adverse effects. The findings provide a foundation for the development of more effective and safer treatment strategies for osteosarcoma.

Future studies should focus on validating these in vitro results through in vivo experiments and exploring the detailed molecular mechanisms involved. It will also be important to evaluate the effects of varying treatment durations and to investigate the role of Hes in combination with other chemotherapeutic agents. These steps will be essential for translating the current findings into clinically applicable therapies.

## Figures and Tables

**Table 1:** Primer Sequences Used for Quantitative Real-Time PCR Analysis of Target Genes.

**Bax: F**: TTCATCCAGGATCGAGCAGA, R: GCAAAGTAGAAGGCAACG
**Bcl-2: F**: ATGTGTGTGGAGAGCGTCAA, R: ACAGTTCCACAAAGGCATCC
**Caspase-3: F**: GGTATTGAGACAGACAGTGG, R: CATGGGATCTGTTTCTTTGC
**Survivin: F**: ACCGCATCTCTACATTCAAG, R: CAAGTCTGGCTCGTTCTC
**β-Actin: F**: CCTCTGAACCCTAAGGCCAAC, R: TGCCACAGGATTCCATACCC
**GAPDH: F**: CGGAGTCAACGGATTTGGTCGTAT, R: GCCTTCTCCATGGTGGTGAAGAC

**Table 2:** Combination Index (CI) analysis of Hes and Cis in U2OS cells and interpretation of interaction effects.

Agents	Method/Model	CI Value	Effect Interpretation
Hes + Cis	Chou–Talalay Model	0.55 (0.44–0.66)	Moderate synergism
Hes + Cis	Bliss Independence Model	0.65 (0.46–0.84)	Moderate synergism
Hes + Cis	Loewe Additivity Model	0.52 (0.37–0.67)	Moderate to strong synergism

## Data Availability

All details about the study can be obtained from the corresponding author.
